# Restraint stress effects on glutamate signaling protein levels in the rats’ frontal cortex: Does β1 adrenoceptor activity matter?

**DOI:** 10.3389/fphar.2024.1451895

**Published:** 2025-01-06

**Authors:** Agnieszka Zelek-Molik, Anna Gądek-Michalska, Michał Wilczkowski, Adam Bielawski, Katarzyna Maziarz, Grzegorz Kreiner, Irena Nalepa

**Affiliations:** ^1^ Department of Brain Biochemistry, Maj Institute of Pharmacology, Polish Academy of Sciences, Krakow, Poland; ^2^ Department of Physiology, Maj Institute of Pharmacology, Polish Academy of Sciences, Krakow, Poland

**Keywords:** adrenocorticotropic hormone, betaxolol, elevated plus maze, frontal cortex, Fyn kinase, restraint stress, glutamate receptors, novel object recognition

## Abstract

**Introduction:**

Stress-evoked dysfunctions of the frontal cortex (FC) are correlated with changes in the functioning of the glutamatergic system, and evidence demonstrates that noradrenergic transmission is an important regulator of this process. In the current study, we adopted a restraint stress (RS) model in male Wistar rats to investigate whether the blockade of β1 adrenergic receptors (β1AR) with betaxolol (BET) in stressed animals influences the body’s stress response and the expression of selected signaling proteins in the medial prefrontal cortex (mPFC).

**Methods:**

The study was divided into two parts. In the first part, rats were exposed to RS for 3, 7, or 14 days, and the expression of glutamate signaling proteins (p(S845)/t GluA1, p(Y1472)/t GluN2B, VGLUT1, and VGLUT2) in the FC was analyzed to determine the optimal RS duration for studying the mechanisms of hypofrontality. In the second part, rats were exposed to RS for 14 days, and BET (5 mg/kg, p. o.) was administered during the last 8 days immediately after RS. The body’s stress reaction was assessed by analyzing body weight and blood levels of adrenocorticotropic hormone (ACTH) and corticosterone (CORT). Behavioral responses were evaluated using the novel object recognition (NOR) and elevated plus maze (EPM) tests. The impact of RS and BET on the expression of p(Y530)/t Fyn and p (S133)/t CREB in the mPFC was measured via Western blotting.

**Results and Discussion:**

The first part of the study demonstrated a decreased level of glutamate receptors in rats exposed to 14 days of RS, following an initial increase observed after 7 days of RS. Results from the second part revealed that chronic RS reduced body weight, impaired recognition memory in the NOR test, augmented blood levels of ACTH, and increased the expression of p(Y530) Fyn in the mPFC. However, β1AR blockade did not alter the effects of RS on weight gain, cognitive function, or the expression of p(Y530) Fyn. β1AR blockade normalized only the blood concentration of ACTH. These results suggest that decreased Fyn kinase activity, indicated by phosphorylation at Y530, underlies the stress-evoked downregulation of GluN2B in the FC in a manner independent of β1AR activity.

## 1 Introduction

During stress reactions, two main body–brain systems are activated sequentially, namely, the autonomic nervous system and the hypothalamic–pituitary–adrenal axis (HPA), leading to an increase in the blood level of glucocorticoids (for details, see McEwen, 2007; Joëls et al., 2012). Through circulating glucocorticoids, the HPA axis mobilizes energy reserves necessary to deal with the extended presence of a stressor or its anticipation (Herman et al., 2016), which is effectively regulated via a negative feedback mechanism (Flak et al., 2012; Myers et al., 2012). Repeated exposure to stress may lead to an unadaptable stress response and the development of psychiatric disorders. A feature of the pathological stress response is reduced frontal cortex (FC) activity (hypofrontality), which has been observed in both clinical (Mayberg et al., 2005; Kamp et al., 2016) and preclinical studies ( Bobula et al., 2011; Chen et al., 2013).

The function of the FC highly depends on the effectiveness of glutamate intracellular signaling (McGinty et al., 2015; Musazzi et al., 2015; Zelek-Molik et al., 2021), which is generally upregulated after acute and short-term exposure to stress but downregulated after chronic stress (see Musazzi et al., 2015). However, the dynamics of these changes and the underlying mechanisms remain elusive.

The activity of glutamate receptors depends on their presence at the neuronal membrane, which is stabilized by the phosphorylation of tyrosine residues at the intracellular C-terminal tail of GluN2B, GluA1 subunits, and the mGluR1/5 dimer. Tyrosine phosphorylation of glutamate receptors in the FC is catalyzed mainly by a family of non-receptor tyrosine kinases, including kinase Fyn (for details, see Mao and Wang, 2016). Interestingly, pharmacologically decreased activity of cortical Fyn in mice is accompanied by BDNF downregulation and depressive-like behavior (Kulikova and Kulikov, 2017).

Noradrenaline (NE) is an important regulator of glutamate receptor activity and cortical function. The noradrenergic system is known to be overactivated in stress pathologies (Birnbaum et al., 1999; Ramos et al., 2005; Joyce et al., 2024). NE signaling is mediated by three types of metabotropic receptors: α2 adrenergic receptors (α2AR), α1 adrenergic receptors (α1AR), and β adrenergic receptors (βAR). It has been shown that NE has the lowest affinity for βAR (740 nM) (Ramos and Arnsten, 2007). Among all βAR, the β1AR is the densest subtype present in the FC (Ramos et al., 2005; Paschalis et al., 2009). We hypothesized that the high NE concentration released during stress reactions in the medial prefrontal cortex (mPFC) through β1AR stimulation may affect the activity of glutamatergic receptors.

We tested our hypothesis using the procedure of restraint stress (RS) in rats, which is commonly used to model stress-related psychiatric disorders (Musazzi et al., 2015). However, during the repeated exposure of animals to the same (homotypic) stressor, including RS, processes of homeostatic adaptation began to develop (Martí and Armario, 1998; Girotti et al., 2006; Gądek-Michalska et al., 2011; Gądek-Michalska et al., 2012; Zelek-Molik et al., 2021), whereby the negative behavioral and physiological consequences of stress are not detected and possibly masked to ensure energy homeostasis (see Herman et al., 2016).

**FIGURE 1:**
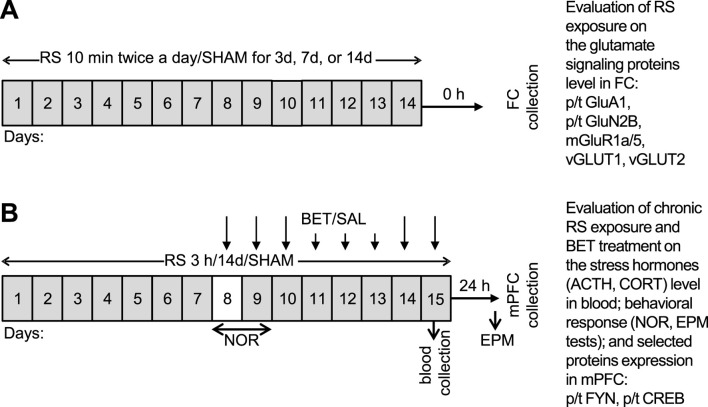
Schedule of experimental procedures undertaken in two parts of the study. **(A)** Mild RS applied 10 min twice a day for 3, 7, or 14 days, adopted to evaluate whether the mild RS exposure develops in FC of rats’ bidirectional effect on the expression of glutamate signaling proteins and therefore can serve as a model of unadaptable stress. The following groups were generated in **(A)** control: RS 3d, RS 7d, and RS14d. **(B)** Regular RS procedure applied for 3 h daily for 14 days together with BET (5 mg/kg/po) treatment during the last 8 days of experiment to evaluate the body stress reaction, recognition memory, anxiety behavior, and indicated protein expression in medial FC (mPFC) after chronic RS and BET treatment. To avoid acute disturbances during familiarization and recognition, rats had 1 day break in RS/SHAM application on experimental day 8 (indicated by the white box), whereas on day 9, the NOR test was performed before the RS session. The following groups were generated in **(B)** SHAM/SAL, RS/SAL, SHAM/BET, and RS/BET.

The aim of the current study was twofold. First, we aimed to check the impact of different durations of exposure to RS (3, 7, and 14 days) on the expression of glutamate signaling proteins, namely, p/t GluA1, p/t GluN2B, and mGluR1a/5, and vesicular glutamate transporters (VGLUT): VGLUT1 and VGLUT2 within the FC of rats. Second, after finding that 14 days is an efficient RS duration to develop hypofrontality, we investigated the impact of chronic RS on cognitive functions using novel object recognition (NOR) and elevated plus maze (EPM) behavioral tests. In biochemical studies, we tested the influence of chronic RS on the expression of p(Y530)Fyn, Fyn, p(S133)CREB, and CREB in the mPFC to identify which signaling pathway could be involved in RS-evoked maladaptation. Additionally, we assessed the therapeutic potential of β1AR blockade using betaxolol (BET, 5 mg/kg, p. o.) to alleviate behavioral and biochemical stress effects. The experimental design alongside the undertaken aims is depicted in Figure 1.

## 2 Materials and methods

### 2.1 Animals

Experiments were conducted on male Wistar rats purchased from Charles River, Germany. The animals were 6 weeks old, weighing approximately 164 g at the beginning of the experiments. They were housed in groups of 4–5 with unlimited access to commercial food and tap water in standard rat cages (UNO Housing, Zevenaar, Netherlands), except during the time of RS, which was administered during the light phase. The following standard laboratory conditions were maintained in the animal room: an artificial 12-h light/dark cycle (lights on from 7 a.m. to 7 p.m.) and a constant temperature of 22°C ± 2 °C. Before the onset of the experiments, the animals were allowed a 1-week habituation period. All procedures were approved by the Local Ethical Commission for Animal Experiments at the Maj Institute of Pharmacology, Polish Academy of Sciences, in Krakow (Permit No. 120/2018, dated 15/03/2018, 168/2018, and 10/05/2018) and fulfilled the requirements of EU Directive 2010/63/EU on the protection of animals used for scientific purposes.

### 2.2 Experimental design

Two parts of experiments on two cohorts of animals were performed to obtain the data presented in this manuscript. The initial part served to evaluate the effects of different durations of RS exposure (3, 7, and 14 days) on the expression of selected glutamate signaling molecules (p/t GluA1, p/t GluN2B, mGluR1a/5, VGLUT1, and VGLUT2) in the FC (Figure 1A). In the second part of the experiment (Figure 1B), rats underwent 14 days of daily RS sessions, and half of them were treated with BET during the last 8 days of experiment to assess whether β1AR blockade could modulate RS effects on the measured behavioral and biochemical parameters. In the behavioral study, NOR and EPM tests were performed to assess recognition memory and anxiety, respectively. NOR was conducted 24 h after 7 days of RS exposure and a single BET application. EPM was performed 24 h after 14 days of RS exposure, with chronic BET application administered during the second week of treatment, immediately after RS. On the 8th experimental day, when RS was not applied to avoid disturbances in NOR training, BET was administered 2 h before the NOR test. In biochemical studies, we assessed the body’s stress reaction by comparing adrenocorticotropin (ACTH) and corticosterone (CORT) levels among the experimental groups. Moreover, we measured the impact of RS and BET on the expression of p/t Fyn and p/t CREB proteins in the mPFC. Finally, we performed the qualitative immunofluorescence analysis of β1AR and GluA1, as well as colocalization analysis of β1AR–GluA1, to identify FC regions where these two receptors might interact.

### 2.3 Stress procedures

Two validated protocols for RS were adopted (Gadek-Michalska et al., 2015; Kusek et al., 2017; Rafa-Zabłocka et al., 2021). For the assessment of the timeline of glutamate signaling changes in FC rats, RS was conducted in metal tubes (diameter: 55 mm) for 10 min twice a day, for 3, 7, or 14 days. The control groups consisted of naive animals that remained in their home cages. Details of this procedure were described elsewhere (Gadek-Michalska et al., 2015). To assess the therapeutic impact of β1AR blockade on RS-evoked behavioral and biochemical parameters, the rats were placed in perforated plastic tubes (6.5 cm inner diameter) of adjustable length. The restraint allowed breathing and limited movements of the head and limbs. The RS procedure lasted 3 h daily for 14 days. After each stress session, the animals were removed from the restrainers and returned to their home cages. Control (SHAM) animals remained in their home cages during the stress sessions. Details of this procedure were described elsewhere (Rafa-Zabłocka et al., 2021). To monitor the rats’ wellbeing and assess their stress response, body weight was recorded daily.

### 2.4 Betaxolol treatment protocol

To assess the role of β1AR in the studied mechanism and to analyze the therapeutic effect of its inhibition, rats were treated with BET, which is a specific antagonist of β1AR used clinically to treat hypertension and is shown to alleviate anxiety in stress models (Rudoy and Van Bockstaele, 2007). BET is a potentially effective tool for modulating brain functions impaired by stress due to its unique pharmacokinetic properties. It is a long-acting β1AR blocker with no intrinsic sympathomimetic activity, and it easily penetrates the blood–brain barrier (Ramos and Arnsten, 2007). In the second week of the RS procedure, treatment with BET (5 mg/kg, p. o.) (Alcon, Fort Worth, TX, United States) or 0.9% NaCl (0.5 mL/rat, p. o.) was introduced immediately after stress. Rats were treated for 8 consecutive days, from the 8th to the 15th experimental day. The dose and route of treatment were chosen based on the literature (Rudoy and Van Bockstaele, 2007) and our previous data (Rafa-Zabłocka et al., 2021).

### 2.5 Novel object recognition

The test consisted of two 5-min trials separated by a 1-day intertrial interval and was performed as previously described (Piotrowska et al., 2024). During the first trial (familiarization, T1), two identical objects were placed in the opposite corners, approximately 10 cm from the walls of the open field. In the second trial (T2, recognition), one of the objects was replaced with a novel object. Animals were returned to their home cage after T1 and T2. The height of the objects was comparable (∼12 cm), and they were heavy enough to prevent displacement by the animals. The location of the novel object in the recognition trial was randomly assigned for each rat. Rats spending less than 5 s exploring the two objects during the trial were eliminated from the study. The exploration time of the objects was measured using the video tracking software EthoVision XT8 (Noldus, the Netherlands). Based on the exploration time of the two objects, a discrimination index was calculated as the time spent exploring the novel object minus the time spent exploring the familiar object.

### 2.6 Elevated plus maze

The apparatus for the elevated plus maze (EPM), made of Plexiglas and elevated to a height of 50 cm, consisted of two open arms (40 × 12 cm) and two closed arms (40 × 12 × 20 cm) arranged at 90° angles to each other, extending from a central platform (12 × 12 cm). The experiment was conducted under low-intensity light (30 Lux). Each rat was placed on the central platform of the maze facing an open arm. A single trial lasted for 5 min and was performed 23 h after the last BET or SAL treatment. Time spent in the open and closed arms, as well as the number of visits to the open and closed arms, was recorded using the video tracking software EthoVision XT8 (Noldus, the Netherlands). Time spent in the open arms and the number of open arm visits served as measures of anxiety.

### 2.7 Brain tissue sample and blood collection

Rats dedicated to checking the profile of changes in the expression of glutamate signaling elements in the FC were decapitated immediately (0 h after the last stress session), and the brains were rapidly removed from the skulls. The FC (frontal part of the brain without the olfactory bulb, extending to 2.7 mm rostral to bregma) was excised on an ice-cold glass plate. During the last RS session, 0.5 mL of blood was collected from the tail veins of rats and placed in EDTA-coated tubes. These rats were decapitated the next day (24 h after the last RS and BET), and their brains were rapidly removed. The mPFC was dissected from 2 mm coronal slices (AP 4.7–2.7 mm rostral to bregma) using a rat brain matrix (Braintree Scientific, MA, United States), as described previously (Sun et al., 2014b). Isolated brain structures were immediately frozen on dry ice and stored at −80°C until assayed. For qualitative immunohistochemistry analysis, the whole brain from a decapitated rat in the SHAM/SAL group was immersed in 4% paraformaldehyde for further processing.

### 2.8 ELISA analysis of CORT and ACTH levels

Plasma from blood samples was isolated according to a previously described protocol (Zelek-Molik et al., 2021). In brief, blood samples were centrifuged at 3,000 × g for 15 min at 4 °C, and the plasma was then transferred to new 1.5-mL collection tubes and stored at −20 °C. CORT and ACTH concentrations were determined using an enzyme-linked immunosorbent assay (ELISA) method with commercially available Rat Corticosterone ELISA and Rat Adrenocorticotropic Hormone ELISA kits (Bioassay Technology Laboratory, Shanghai, China). The immunoenzymatic reaction was prepared and developed according to the manufacturer’s instructions. Serum samples, tested in duplicates, were diluted fivefold before carrying out the assays. The absorbance was measured at 450 nm using a plate reader (Synergy MX, BioTek, Winooski, VT, United States). Hormone concentrations were calculated from standard curves fitted using four-parameter logistic equations in GraphPad Prism 5.0 (GraphPad Software, San Diego, CA, United States). The sensitivity of ELISA assays was 0.24 ng/mL for CORT and 2.49 pg/mL for ACTH.

### 2.9 Immunoblotting

The procedure of immunoblotting was described previously (Zelek-Molik et al., 2021). In brief, total protein extraction was performed using radioimmunoprecipitation assay (RIPA) buffer (MilliporeSigma, Burlington, MA, United States). Equal amounts of protein extracts were diluted with a loading buffer containing an inclusion body solubilization buffer (G-Biosciences, Saint Louis, MO, United States) and a reducing agent, 1% 2-mercaptoethanol. The samples were then denatured at 45 °C for 30 min. Denatured samples were run on SDS-PAGE gels and transferred onto nitrocellulose membranes. The membranes were blocked using 5% nonfat dry milk in Tris-buffered saline containing 0.1% Tween-20 (TBST; pH 7.6) for 1 h at room temperature and then incubated with specific primary antibodies. All studied glutamatergic receptors were assessed on the same membranes. After staining with Ponceau S, the membranes were horizontally cut at the 150 kDa level. Proteins—mGluR1/5 dimer (250 kDa) and p- and total GluN2B (180 kDa)—were assessed on the upper section, whereas GluA1 (106 kDa) was assessed on the bottom section. The expression of glutamate transporters, p/t Fyn, and p/t CREB was analyzed on separate gels. Following overnight incubation at 4 °C with primary antibodies and three washes using the blocking solution, the membranes were incubated with appropriate secondary antibodies for 1 h at room temperature, followed by three additional washes with TBST. Antibody binding was detected using an enhanced chemiluminescence kit (ECL Plus 32106, Thermo Fisher Scientific, Waltham, MA, United States). Equal protein loading was further confirmed by probing using anti-calnexin antiserum (1:5,000; ADI-SPA-865-F, Enzo Life Sciences, Farmingdale, NY, United States) or anti-β-actin antiserum (1:5,000; A5441, MilliporeSigma, Burlington, MA, United States). The following antibodies were used in the experiment: p(Y1472)GluN2B (1:1,000, M2442, MilliporeSigma, Burlington, MA, United States), GluN2B (1:1,000, 610416, BD Biosciences, San Jose, CA, United States), mGluR5/1a (1:2000, 2032-mGluR5/1a, PhosphoSolution, Aurora, CO, United States), GluA1 (1:2000, ab31232, Abcam, United Kingdom), VGLUT1 (1:1,000, MAB5502, MilliporeSigma, Burlington, MA, United States), VGLUT2 (1:1,000, D7D2H, Cell Signaling Technology, Danvers, MA, United States), p (Y530)Fyn (1:1,000, ab182661, Abcam, United Kingdom), Fyn (1:1,000, ab125016, Abcam, United Kingdom), p (S133)CREB (1:1,000, 06–519, MilliporeSigma, Burlington, MA, United States), and CREB (1:5,000, Cell Signaling Technology, Danvers, MA, United States). All Western blot analyses were performed at least twice to confirm the results. The chemiluminescence of specific signals was visualized using a multi-application gel imaging system, and the immunoreactive bands were quantified using an image analyzer (Multi Gauge V3.0, Fujifilm, Tokyo, Japan).

### 2.10 Immunohistochemistry

Rat brains were fixed in 4% paraformaldehyde overnight and processed as described previously (Bielawski et al., 2023), with further modifications. The fixed brains were embedded in paraffin and coronally sectioned at a thickness of 7 µm using a rotary microtome (Leica, Wetzlar, Germany). For GluA1 and β1AR staining, selected sections containing the mPFC and M1/M2 regions of the FC (bregma +3.0 mm) were subjected to deparaffinization and antigen retrieval using the microwave method with citrate buffer. Subsequently, these sections were immersed in a blocking solution consisting of 5% normal goat serum (S-1000–20; Vector Laboratories, CA, United States) dissolved in PBS. The localization of GluA1 and β1AR was confirmed by labeling with a mouse anti-GluA1 antibody (1:100, Sigma-Aldrich, St. Louis, Missouri, United States) and a rabbit anti-β1AR antibody (1:250, Abcam, United Kingdom). The primary antibodies bound to antigens were visualized using anti-mouse Alexa-488 and anti-rabbit Alexa-594 secondary antibodies (1:400; Molecular Probes, Eugene, OR, United States). Stained sections were examined and photographed in the widefield mode using a Leica TCS SP8 microscope.

### 2.11 Statistical analysis

**FIGURE 2:**
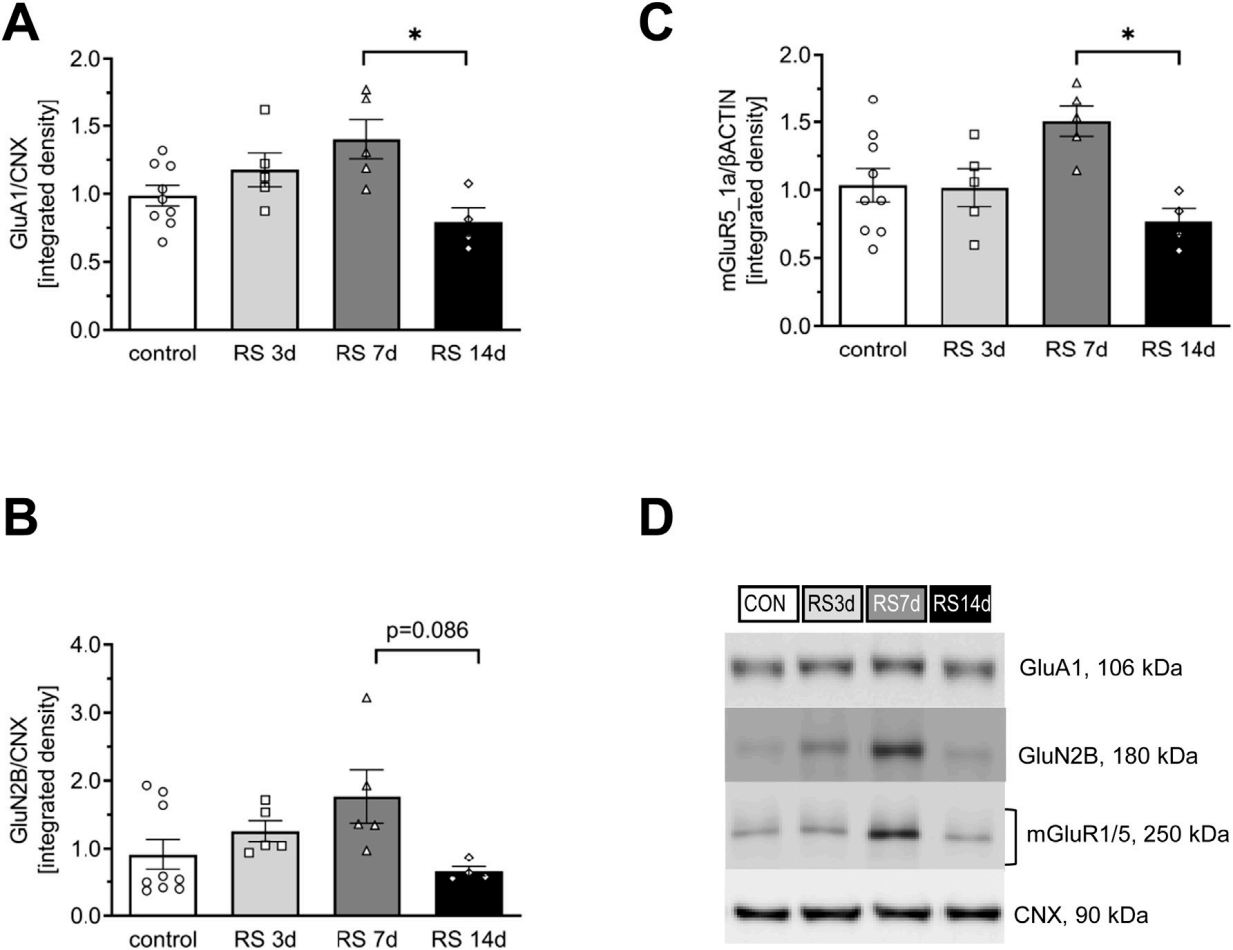
Influence of RS and its duration on the expression of selected glutamate receptors in the rat FC. **(A)** The expression of the GluA1 subunit of AMPA-R. **(B)** The expression of the GluN2B subunit of NMDA-R. **(C)** The expression of mGluR5/1a. **(D)** Representative immunoblots that illustrate GluA1, GluN2B, and mGluR5/1a expression in the FC of experimental groups. Data were calculated as control percentages and are expressed as mean ± SEM, N = 4–9/group. In **(A)** [F (3, 19) = 4,85; *p* = 0.011, in **(B)** [F (3, 19) = 2.99; *p* = 0.057], and in **(C)** [F (3, 19) = 4.57; *p* = 0.014]; *p < 0.05 (unequal N HSD).

**FIGURE 3:**
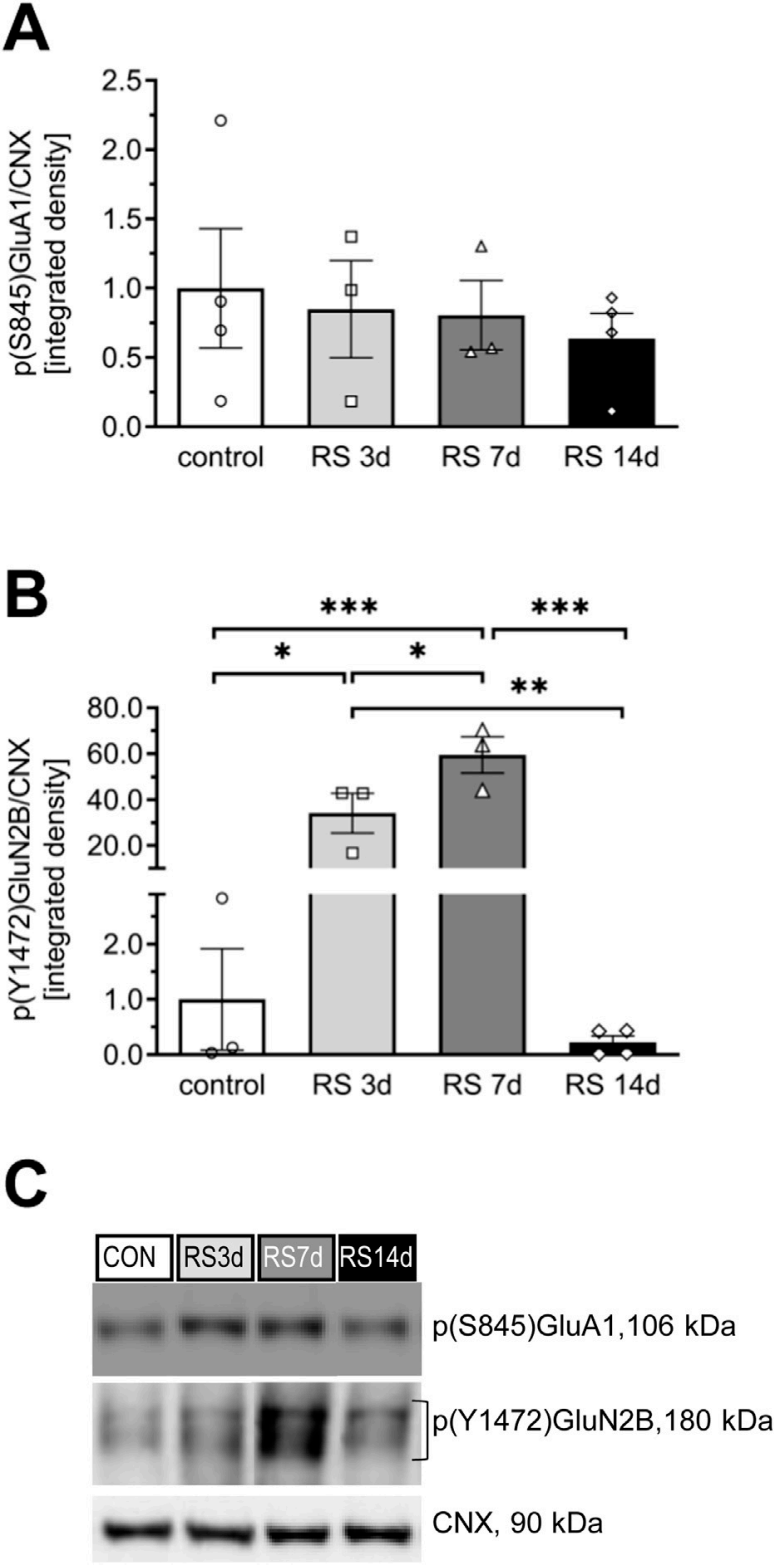
Influence of RS and its duration on the phosphorylation level of the GluA1 and GluN2B subunits in rat FC. **(A)** The expression of p(S845)GluA1. **(B)** The expression of p(Y1472)GluN2B. **(C)** Representative immunoblots that illustrate GluA1 and GluN2B phosphorylation levels in the FC of experimental groups. The phosphorylated GluA1 and GluN2B proteins were assessed on the same membrane as total proteins, and both were normalized to the same loading control. Therefore, the mutual representative blot for CNX is visualized in both Figures 2D, 3C. Results are expressed as a mean ± SEM, N = 3–4/group. In **(A)** [F (3, 10) = 0.24; *p* = 0.868] and in **(B)** [F (3, 9) = 28.35; *p* < 0.0001]. ***p< 0.001, **p< 0.01, and *p< 0.05 indicated in graph *p* = 0.08 (unequal N HSD).

All values are presented as percentages of controls and are expressed as the mean ± standard error of the mean (SEM), with group sizes ranging from N = 3 to 9 rats. The small sample size (N = 3–4) applied to the Western blot results shown in Figure 3, which were designed to verify whether the changes observed in Figure 2 of the manuscript are localized to the cell membrane. Thus, the small sample size should not interfere with the overall conclusions of the article. Statistical analyses were performed using Statistica 10 (Round Rock, TX, United States). Data were evaluated using one- or two-way analysis of variance (ANOVA), followed by a *post hoc* test (unequal N HSD or Fisher LSD) where appropriate. A significance level of *p* < 0.05 was considered indicative of a significant effect. Within-group comparisons of novel vs. familiar object recognition in the NOR test were conducted using a t-test for independent samples.

## 3 Results

### 3.1 Bidirectional profiles of changes evoked by RS on the level of glutamate receptor proteins in the rat FC

#### 3.1.1 GluA1 protein

In the case of total GluA1, one-way ANOVA revealed a significant difference among the analyzed groups [F (3, 19) = 4.85; *p* < 0.05]. The *post hoc* analysis of the influence of different durations of restraint stress on the expression of GluA1 in the rat FC showed a gradual increase in protein levels in the RS3d and RS7d groups compared to the control group (by 18% and 40%, respectively). The augmented GluA1 level in the RS7d group was on the borderline of statistical significance compared to the control (*p* = 0.08). Two weeks of RS application resulted in a 60% decrease in GluA1 levels compared to the RS7d group (and a 21% insignificant decrease compared to the control) (Figures 2A,D).

No significant changes were observed among groups in the phosphorylation level of p(S845)GluA1 [F (3, 10) = 0.24; *p* = 0.87] (Figures 3A,C).

#### 3.1.2 GluN2B protein

In the case of total GluN2B, one-way ANOVA revealed an effect on the borderline of statistical significance [F (3, 19) = 2.99; *p* = 0.056]. Restraint stress initially evoked a gradual increase in GluN2B levels in the RS3d and RS7d groups compared to the control group, by 38% and 94%, respectively; however, this effect was statistically insignificant. Two weeks of RS resulted in a 63% decrease in GluN2B expression compared to the RS7d group (*p* = 0.086) and a 27% insignificant decrease compared to the control group (Figures 2B,D). A more pronounced effect was observed for the p(Y1472)GluN2B level [F (3, 9) = 28.35; *p* < 0.0001], which gradually increased in the RS3d and RS7d groups compared to the control group by approximately 30-fold (*p* < 0.05) and 60-fold (*p* < 0.01), respectively. After 14 days of RS, the p(Y1472)GluN2B level decreased below the control level (Figures 3B,C).

#### 3.1.3 mGluR5/1a protein

One-way ANOVA revealed significant differences among the analyzed groups [F (3, 19) = 4.57; *p* < 0.05]. Restraint stress after 3 days of treatment did not alter the level of mGluR5/1a. An insignificant 51% increase was observed in the RS7d group compared to the control group. However, after 14 days of RS, mGluR5/1a expression was significantly decreased by 49% compared to the RS7d group and exhibited an insignificant 25% decrease compared to the control group (Figures 2C,D).

### 3.2 Profile of changes evoked by RS on the level of glutamate transporters in the rat FC

**FIGURE 4:**
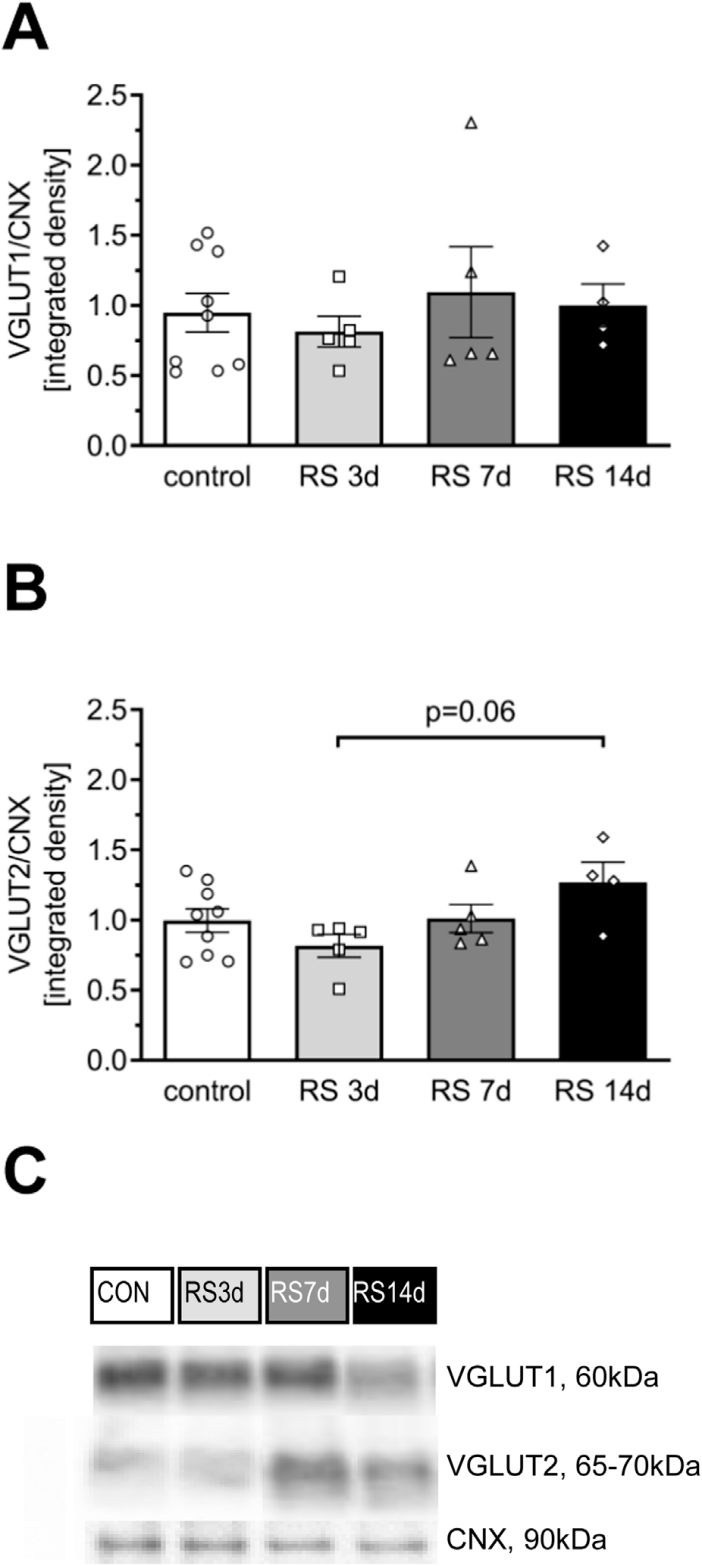
Influence of RS and its duration on the expression of selected glutamate transporters in the rat FC. **(A)** The expression of VGLUT1. **(B)** The expression of VGLUT2. **(C)** Representative immunoblots that illustrate VGLUT expression in the FC of experimental groups. Results are expressed as a mean ± SEM, N = 4–9/group. In **(A)** [F (3, 19) = 0.32; *p* = 0.807] and **(B)** [F (3, 19) = 2.66; *p* = 0.077] indicated in graph *p* = 0.06 (unequal N HSD).

There were no significant differences among treatment groups in the level of VGLUT1 [F (3, 19) = 0.32; *p* = 0.81] (Figures 4A,C). One-way ANOVA for VGLUT2 expression revealed an effect on the borderline of statistical significance [F (3, 19) = 2.66; *p* = 0.077]. The *post hoc* analysis indicated that after a slight decrease in the RS3d group (by 19% compared to the control), VGLUT2 expression in the RS7d group returned to the control level, whereas in the RS14d group, it was insignificantly increased by 28% compared to the control. The increase in VGLUT2 expression in the RS14d group was on the borderline of statistical significance compared to the RS3d group (by 54%; *p* = 0.06) (Figures 4B,C).

### 3.3 Rat body response to prolonged RS exposure and BET treatment

#### 3.3.1 Body weight

**FIGURE 5:**
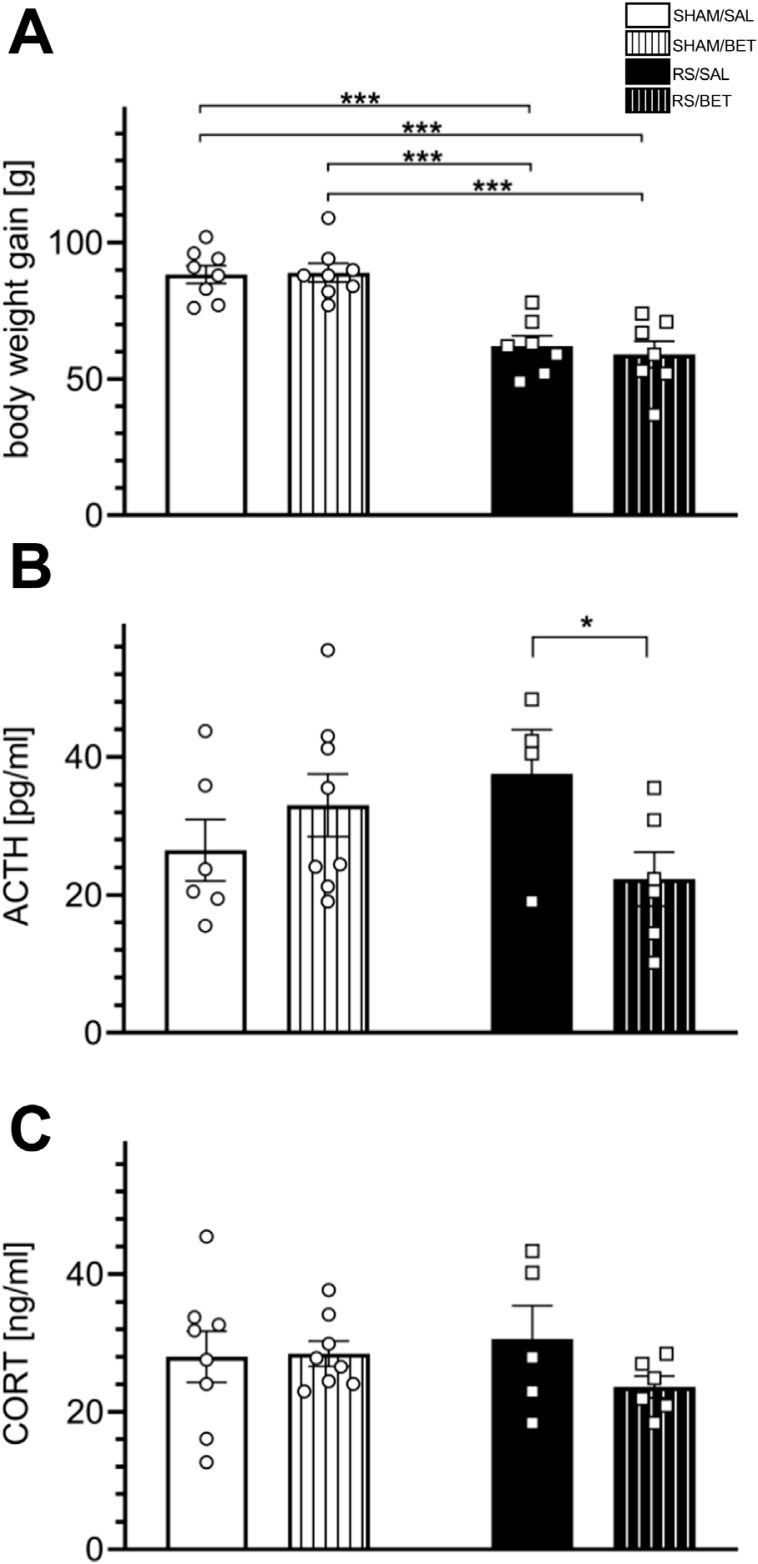
Body stress reaction to chronic RS and BET treatment. **(A)** Body weight gain during experimental procedures, **(B)** the concentration of ACTH, and **(C)** the concentration CORT. Stress hormones were assessed in plasma isolated from tail vein blood taken during the last RS session. White bars correspond to SHAM, black to RS pretreatment, and bars with patterns correspond to BET posttreatment. BET (5 mg/kg/po/14 days) applied immediately after RS. Results are expressed as a mean ± SEM; N = 4–8/group. In **(A)** RS [F (1,26) = 53.97; p< 0.001], BET [F (1,26) = 0.096; *p* = 0.76], and RS × BET interaction [F (1,26) = 0.22; *p* = 0.64]. In **(B)** RS effect [F (1,19) = 0.158; *p* = 0.69], BET effect [F (1,19) = 0.745; *p* = 0.20], and RS × BET interaction [F (1,20) = 4.238; *p* = 0.05]. In **(C)** RS effect [F (1,23) = 0.131; *p* = 0.72], BET [F (1,23) = 1.064; *p* = 0.31], and RS×BET effect [F (1,23) = 1.374; *p* = 0.25]. ***p< 0.001 (unequal N HSD).

The initial body weight ±SEM of the animal groups analyzed in the study is as follows: SHAM/SAL – 167 ± 2.52 g, n = 8; SHAM/BET – 164 ± 3.31 g, n = 8; RS/SAL – 161 ± 3.36 g, n = 8; and RS/BET – 164 ± 3.31 g, n = 8. A two-way ANOVA revealed a significant effect of RS on body weight gain during the entire RS procedure [F (1, 26) = 53.97; *p* < 0.001]. There was no significant effect of BET [F (1, 26) = 0.096; *p* = 0.76] or the RS × BET interaction [F (1, 26) = 0.22; *p* = 0.64]. The *post hoc* analysis revealed that BET did not affect body weight gain, whereas the RS/SAL and RS/BET groups showed reduced body weight gain compared to the SHAM/SAL group, by 30% and 33%, respectively (Figure 5A).

#### 3.3.2 Plasma level of stress hormones

For ACTH concentration, two-way ANOVA revealed no significant effect of RS [F (1, 19) = 0.158; *p* = 0.69] or BET [F (1, 19) = 0.745; *p* = 0.20]. However, the RS × BET interaction effect was on the borderline of statistical significance [F (1, 20) = 4.238; *p* = 0.05]. A comparison of mean values among groups showed an insignificant increase in ACTH concentration in the RS/SAL group (by 42% compared to SHAM/SAL), which returned to the control level after BET treatment. The ACTH level in the RS/BET group was significantly reduced compared to that in the RS/SAL group (*p* = 0.038; LSD test) (Figure 5B). For CORT concentration, no significant main effects were observed: RS effect [F (1, 23) = 0.131; *p* = 0.72], BET effect [F (1, 23) = 1.064; *p* = 0.31], or RS × BET interaction [F (1, 23) = 1.374; *p* = 0.25] (Figure 5C).

### 3.4 The behavioral response of rats to prolonged RS exposure and BET treatment

#### 3.4.1 Novel object recognition

**FIGURE 6:**
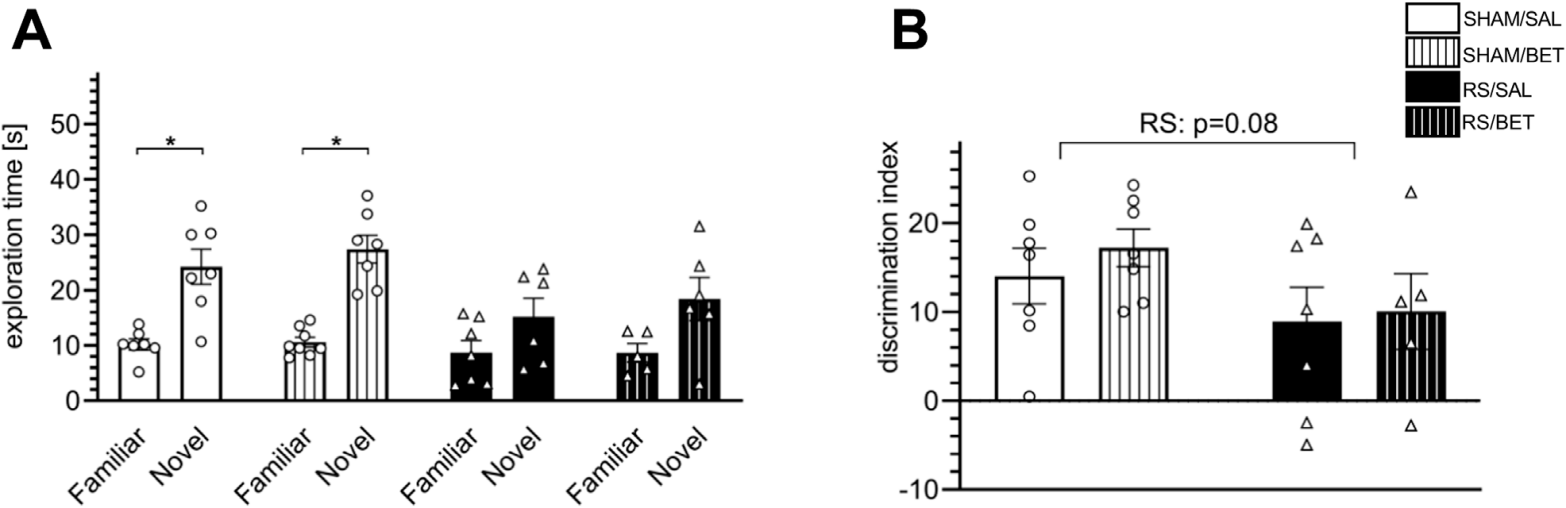
Effects of RS and single BET on the rats’ behavior measured in the NOR test. **(A)** Exploration time of a novel and a familiar object by particular groups in the retention trial (T2). **(B)** Discrimination index (DI) measured as the time spent exploring the novel object minus time spent exploring the familiar object. White bars correspond to SHAM, black to RS pretreatment, and bars with patterns correspond to BET posttreatment; BET (5 mg/kg/po/1×) applied immediately after 7th RS session. Results are expressed as a mean ± SEM. N = 6–8/group. In **(B)** RS [F (1,22) = 3.3100; *p* = 0.08 (p-value indicated in the graph)], BET [F (1,22) = 0.40; *p* = 0.53], and RS × BET interaction [F (1,22) = 0.009; *p* = 0.76]. *p< 0.05 indicates significant difference in time spent exploring the novel object compared with the familiar object (t-test).

To avoid acute disturbances during familiarization and recognition, the rats had a break from the stress session on the training day and until the test trial. Rats in the BET groups received the drug after familiarization. The test trial (T2) was performed only on rats that explored two novel objects in the training trial (T1) with no side preference. In T2, all tested rats in the SHAM groups spent significantly more time exploring the novel object, and a single BET treatment did not alter this preference (Figure 6A). Within-group comparisons showed significant results for SHAM/SAL [t (12) = −4.24; *p* < 0.01] and SHAM/BET [t (13) = −6.62; *p* < 0.001]. In contrast, rats in the RS/SAL group did not show a preference for exploring the novel object [t (11) = −1.67; *p* = 0.13], and the effect in the RS/BET group was also insignificant [t (9) = −2.13; *p* = 0.06]. The analysis of the discrimination index revealed a lower discrimination index in stressed animals. The RS effect was on the borderline of statistical significance [F (1, 22) = 3.31; *p* = 0.08]. No significant effects of BET [F (1, 22) = 0.40; *p* = 0.53] or RS × BET interaction [F (1, 22) = 0.09; *p* = 0.76] on the discrimination index in the NOR test were observed (Figure 6B).

#### 3.4.2 Elevated plus maze

There were no significant effects of RS, BET, or RS × BET interaction on the time spent in the open arms (RS: [F (1, 26) = 0.16; *p* = 0.69], BET: [F (1, 26) = 0.02; *p* = 0.89], and RS × BET: [F (1, 26) = 0.11; *p* = 0.75]) or in the closed arms (RS: [F (1, 26) = 0.01; *p* = 0.94], BET: [F (1, 26) = 0.46; *p* = 0.50], and RS × BET: [F (1, 26) = 0.02; *p* = 0.87]) of the EPM apparatus. Similarly, there were no significant effects on the number of entries into the open arms (RS: [F (1, 26) = 0.39; *p* = 0.84], BET: [F (1, 26) = 0.05; *p* = 0.82], and RS × BET: [F (1, 26) = 1.32; *p* = 0.26]) or the closed arms (RS: [F (1, 26) = 0.12; *p* = 0.73], BET: [F (1, 26) = 0.15; *p* = 0.70], and RS × BET: [F (1, 26) = 0.0006; *p* = 0.98]). The mean time ±SEM spent by all tested animals in the open arms was 70.58 ± 7.40 s, which was more than twofold lower than the time spent in the closed arms (150.94 ± 9.90 s) (t = 6.50, df = 58, *p* < 0.0001, and unpaired t-test) (Supplementary Figure S1).

### 3.5 Effects of the prolonged restraint stress exposure and betaxolol treatment on Fyn and CREB expression and phosphorylation in the rat mPFC

#### 3.5.1 Fyn protein

**FIGURE 7:**
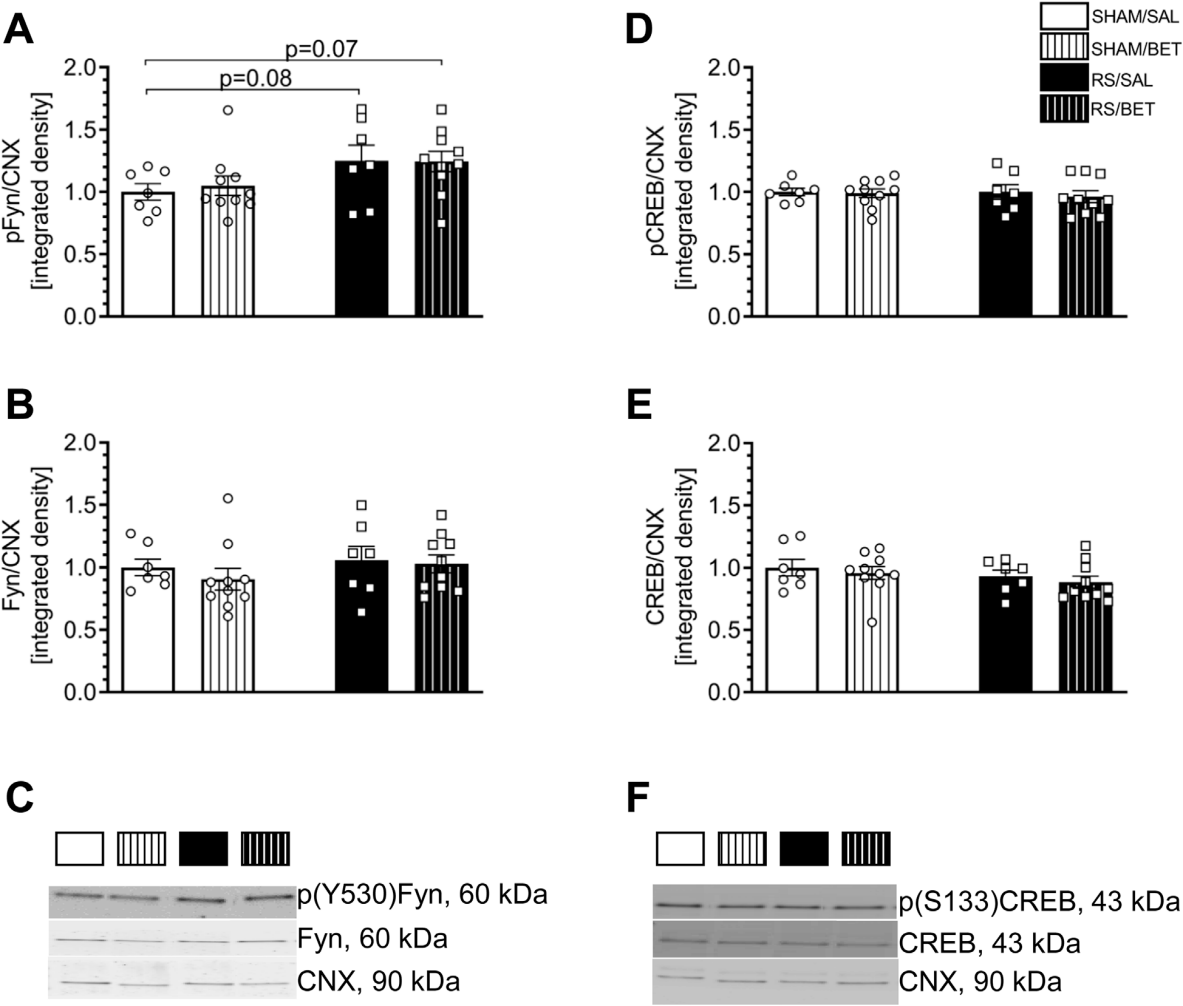
Effects of chronic RS and BET treatment on the phosphorylation and total level of Fyn and CREB proteins in the mPFC of rats measured by 24 h after the last treatment. **(A)** The expression of p (Y530)/Fyn. **(B)** The expression of Fyn. **(C)** Representative immunoblots for p(Y530)Fyn and Fyn. **(D)** The expression of p(S133)CREB. **(E)** The expression of CREB. **(F)** Representative immunoblots for p(S133)CREB and CREB. White bars correspond to SHAM, black to RS pretreatment, and bars with patterns correspond to BET post-treatment. BET (5 mg/kg/po/14 days) applied immediately after RS. Results are expressed as a mean ± SEM. N = 6–8/group. In **(A)** RS [F (1,30) = 6.0012; *p* = 0.02], BET [F (1,30) = 0.06; *p* = 0.81]; and RS × BET interaction [F (1,30) = 0,93; *p* = 0.76]. In **(B)** RS [F (1,30) = 1.07; *p* = 0.31], BET [F (1,30) = 0.05; *p* = 0.48], and RS × BET interaction [F (1,30) = 0.14; *p* = 0.70]. In **(D)** RS [F (1,30) = 0.09; *p* = 0.76], BET [F (1,30) = 0.29; *p* = 0.59], and RS × BET interaction [F (1,30) = 0.09; *p* = 0.80]. In **(E)** RS [F (1,30) = 1.67; *p* = 0.20], BET [F (1,30) = 0.72; *p* = 0.40], and RS × BET interaction [F (1,30) = 0.003; *p* = 0.95]. *p* = 0.08 and *p* = 0.07 values indicated in graph (Fisher LSD test).

Stress augmented the level of p(Y530)Fyn independently of BET treatment. Two-way ANOVA revealed a significant RS effect [F (1, 30) = 6.0012; *p* = 0.02] on the level of p(Y530)Fyn. There were no significant effects of BET [F (1, 30) = 0.06; *p* = 0.81] or RS × BET interaction [F (1, 30) = 0.93; *p* = 0.76]. The *post hoc* Fisher LSD test showed a 25% increase in the phosphorylation level of p(Y530)Fyn in the RS/SAL group (*p* = 0.082) and the RS/BET group (*p* = 0.066) compared to the SHAM/SAL group (Figures 7A,C).

No significant effects of RS [F (1, 30) = 1.07; *p* = 0.31], BET [F (1, 30) = 0.05; *p* = 0.48], or RS × BET interaction [F (1, 30) = 0.14; *p* = 0.70] were observed on the expression of the total Fyn protein (Figures 7B,C).

#### 3.5.2 CREB protein

Neither RS nor BET influenced p(Ser133)CREB or CREB expression in the mPFC. Two-way ANOVA results for p(Ser133)CREB are as follows: RS [F (1, 30) = 0.09; *p* = 0.76], BET [F (1, 30) = 0.29; *p* = 0.59], and RS × BET interaction [F (1, 30) = 0.09; *p* = 0.80] (Figures 7D,F). Similarly, two-way ANOVA results for CREB are as follows: RS [F (1, 30) = 1.67; *p* = 0.20], BET [F (1, 30) = 0.72; *p* = 0.40], and RS × BET interaction [F (1, 30) = 0.003; *p* = 0.95] (Figures 7E,F).

### 3.6 Qualitative comparison of β1AR–GluA1 colocalization in M1/M2 vs. mPFC

**FIGURE 8:**
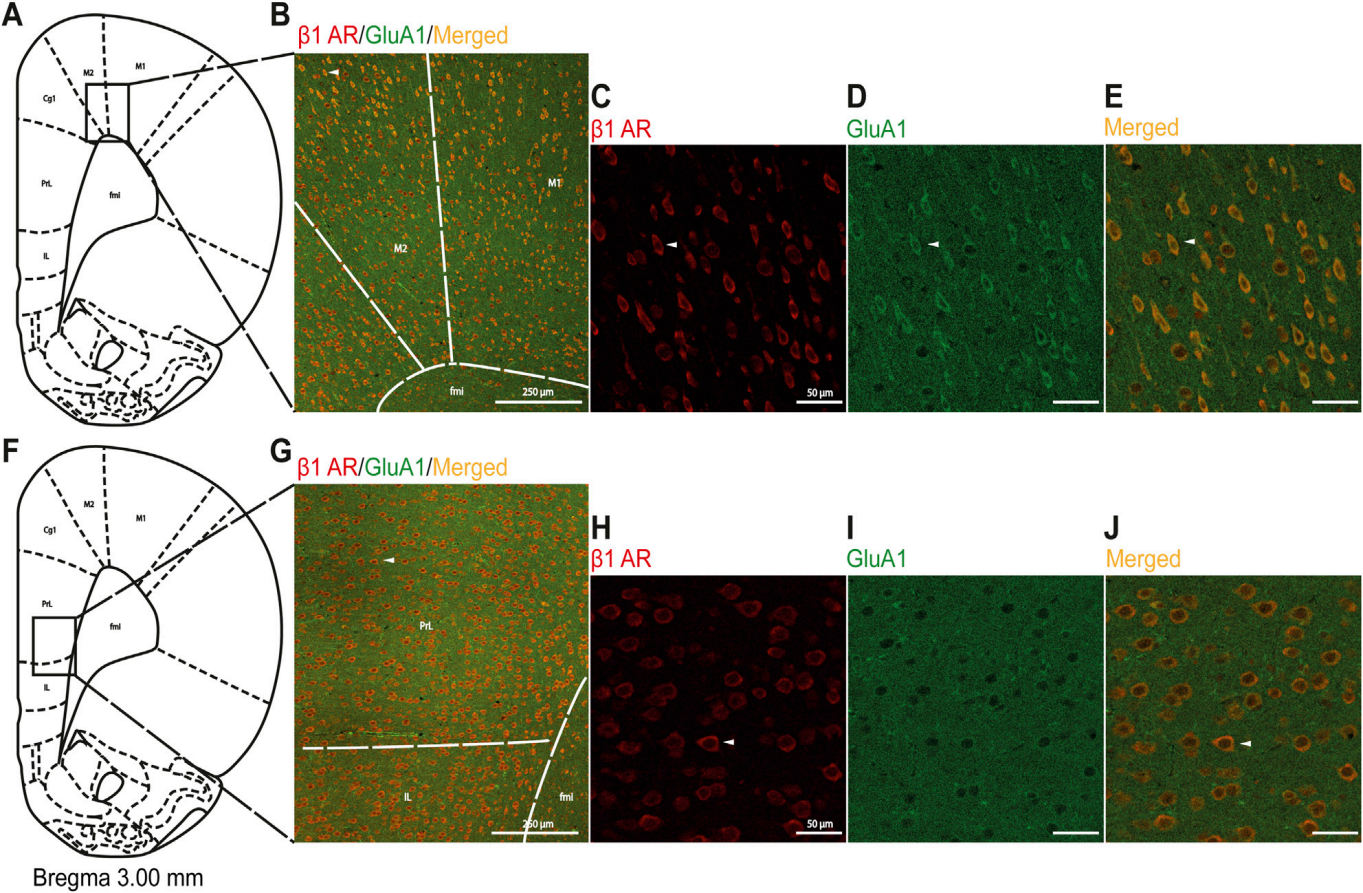
Immunofluorescent qualitative detection of colocalization of the β1AR and GluA1 subunits of AMPA-R in M1/M2 and mPFC regions. **(A)** Schematic illustration of the analyzed area for M1/M2, and **(B)** a representative image with drawn lines indicating borders among adjacent areas. In **(C-E)**, successively presented, representative magnified images with immunofluorescence staining for β1AR (red), GluA1 (green), and merged (orange) in M1/M2. **(F)** Schematic illustration of the analyzed area for mPFC, and **(G)** a representative image with drawn lines indicating borders among adjacent areas. In **(H-J)**, successively presented, representative magnified images with immunofluorescence staining for β1AR (red), GluA1 (green), and merged (orange) in mPFC. Scale bars in **(B)** and **(G)** 250 μm; in **(C-E)** and **(H-J)**: 50 µm. White arrows indicate the same cell at different magnifications in the upper and bottom panels. Abbreviations: Cg1, cingulate cortex; fmi, forceps minor of the corpus callosum; IL, infralimbic cortex; M1, primary motor cortex; M2, secondary motor cortex; PL, prelimbic cortex.

Based on the knowledge of the cooperation between βAR and GluA1, which is necessary to modulate intracellular stress signaling in the hippocampus (Joiner et al., 2010), we performed a colocalization analysis of β1AR with GluA1 to identify loci other than the mPFC within the FC where βAR could modulate glutamatergic signaling. The colocalization of β1AR and GluA1 proteins was more clearly visible in the M1/M2 regions than that in the mPFC (Figure 8).

## 4 Discussion

We demonstrated an increase in the level of glutamate receptors in the FC of rats after 7 days of RS exposure, followed by a decrease observed on the 14th day of RS. Furthermore, we showed that chronic RS impaired recognition memory and elevated the p(Y530)Fyn level in the mPFC in a manner independent of β1AR activity.

### 4.1 Bidirectional profile of RS evoked changes in the glutamate signaling proteins within FC

Preclinical studies utilizing genetic, biochemical, electrophysiological, pharmacological, and lesion techniques have revealed the involvement of glutamate receptors—particularly the GluA1 subunit of α-amino-3-hydroxy-5-methyl-4-isoxazolepropionic acid receptors (AMPA-R), the GluN2B subunit of N-methyl-d-aspartate receptors (NMDA-R), and the mGluR1a/5 dimer of group I metabotropic glutamate receptors (mGluRI)—in stress-affected synaptic functioning of the FC (Lu et al., 2009; Musazzi et al., 2015; Mikics et al., 2017; Zelek-Molik et al., 2021). In the present study, we observed a gradual increase in GluA1 and GluN2B levels in the FC of RS3d and RS7d groups compared to the control group, followed by a decrease in the RS14d group compared to the RS7d group. A similar bidirectional profile of changes was noted for mGluR1a/5. These results confirm the involvement of GluA1, GluN2B, and mGluR1a/5 in the FC as part of the mechanism underlying the stress pathology. The bidirectional profile of glutamate receptor level changes observed in this study aligns with findings from other stress models (for review, see Musazzi et al., 2015; Mayberg et al., 2005). However, the dynamics of stress-evoked changes in glutamate receptor levels observed in our study differed from those reported in the literature. We noted upregulation of GluA1, GluN2B, and mGluR1a/5 after 7 days of RS, whereas others detected augmented levels of glutamate receptors following acute RS (Yuen et al., 2009). A possible explanation for this discrepancy is the less severe RS protocol used in the first part (A) of our experiments (10 min twice daily) compared to the typically applied 2–3 h daily RS. This explanation aligns with the observations of Cristina Rabasa et al., who showed slower adaptation of HPA responses to homotypic stress with lighter intensity (Rabasa et al., 2015). The upregulation of glutamatergic receptor subunits in the FC after acute RS exposure has been associated with improved behavioral performance (Yuen et al., 2009). Assuming that an increased glutamate receptor level in the FC is a biomarker of improved working memory, our results suggest that this facilitation under mild RS conditions (10 min twice daily) persists longer, lasting at least 7 days. Notably, applying the same RS duration but with higher intensity (3 h daily) in the second part (B) of our experiments impaired recognition memory in rats. Following the increase in GluA1, GluN2B, and mGluR1a/5 levels observed in the FC of rats exposed to 7 days of RS, a downregulation was noted after 14 days of RS exposure. It should be noted that the expression of glutamate subunits of ionotropic receptors encompasses not only the membrane fraction but also other intracellular compartments, where GluA1 and GluN2B are synthesized and processed. However, in our study, the effects of RS appeared to be specific to membrane NMDA-Rs containing GluN2B subunits. This conclusion is supported by the observation that RS, in our study, had a similar effect on the phosphorylation level of these subunits, a process that occurs only at the plasma membrane (Goebel-Goody et al., 2012). Chronic RS and CORT administration have been associated with increased field potential amplitudes in the FC (Bobula et al., 2011) and enhanced presynaptic glutamate release (Liu et al., 2020; Modrak et al., 2023). In the present study, we demonstrated an increased level of the presynaptic VGLUT2, likely reflecting the described glutamate release adaptation in the presynaptic elements of stressed synapses within the FC. VGLUT2 is considered a marker of thalamocortical and mesocortical projections (Liguz-Lecznar and Skangiel-Kramska, 2007; Gorelova et al., 2012). Therefore, changes in VGLUT2, but not in VGLUT1, suggest that RS affects glutamatergic inputs from the thalamus or ventral tegmental area. A similar pattern of changes in VGLUT levels was recently observed in a model of chronic psychosocial stress (Zelek-Molik et al., 2021), suggesting that both social and physical stressors affect similar cortical projections. The downregulation of glutamate receptor levels, together with the upregulation of VGLUT2 in the FC observed after 14 days of mild RS exposure, indicates that 14 days is an efficient duration for modeling stress-evoked hypofrontality, even under less severe stress conditions. Consequently, we selected 14 days of RS as an optimal period to further investigate the involvement of Fyn and CREB protein activity in stress pathology mechanisms, as well as to evaluate the therapeutic potential of systemic β1AR blockade applied after 1 week of RS.

### 4.2 Body stress response can be attenuated by β1AR blockade

To evaluate the applicability of the RS procedure as a model for stress-related hypofrontality in the context of potential homeostatic adaptation, we conducted a comprehensive analysis of the physiological and behavioral stress responses after chronic RS exposure. The slower body weight gain observed in stressed animals reflects profound physiological changes induced by prolonged stress exposure. This observation aligns with findings from other studies employing RS protocols (Magariños and McEwen, 1995; Harris et al., 2002; McLaughlin et al., 2007). The RS-induced reduction in body weight gain is unlikely to result from food restrictions during RS sessions, as rats were weighed both before the stress session and after a 21-h period of unrestricted food access. Furthermore, studies monitoring food intake in restrained animals have shown that RS can increase food consumption compared to control groups (Kleen et al., 2006). Thus, the slower weight gain in RS rats likely reflects the physiological effects of an uncontrollable stressor, which is known to influence the FC structure and function and contribute to the development of stress-related psychiatric disorders. In behavioral studies, rats exposed to RS (3 h daily) for 7 days spent a similar amount of time exploring familiar and novel objects in the NOR test, unlike non-stressed control rats. Consequently, the discrimination index was lower in RS rats than that in SHAM groups. This finding corroborates previous studies showing that rats exposed to daily RS for 1–3 weeks exhibit impaired object discrimination (Bowman et al., 2009; Bowman et al., 2022; Yuen et al., 2012; Luine et al., 2017), indicating deficits in visual (non-spatial) memory. However, chronic RS in our study did not induce anxiety in the EPM test, which differed from the results in male rats subjected to more severe RS protocols (6 h daily for 21 or 28 days) (Bowman et al., 2009; Chiba et al., 2012). Taken together, these findings suggest that the RS protocol used in our study (3 h daily for 14 days) is sufficient to impair recognition memory but lacks the intensity to induce anxiety. Analysis of stress hormone levels during the last RS session revealed no changes in CORT levels but an increase in ACTH levels, which was normalized by BET treatment. The absence of increased CORT levels during the 14th RS session aligns with the findings from other chronic RS models (Gądek-Michalska et al., 2013; Kusek et al., 2017) and likely reflects adaptive homeostasis (habituation) to a processive stressor, which is classified as “non-damaging” (Girotti et al., 2006; Davies, 2016). It is noteworthy, however, that the tail veins of RS rats appeared highly shrunken compared to non-stressed controls, making blood collection difficult (personal observation). This finding suggests stress anticipation by RS rats, despite unchanged CORT levels across experimental groups. The absence of elevated CORT levels may indicate a functional negative feedback mechanism that rapidly extinguishes HPA responses to predictable stressors, thereby preserving the capacity to respond appropriately to novel (heterotypic) stressors (Herman et al., 2016). Additionally, ACTH levels, rather than CORT, are considered markers of stressor intensity. Chronic exposure to a strong stressor has been shown to result in sustained ACTH elevation and reduced CORT levels compared to initial acute RS responses (Martí and Armario, 1998). The increased ACTH levels observed in our study suggest that the chronic RS protocol was sufficiently intense to induce prolonged stress anticipation and influence FC structure and function. The secretion of ACTH from the pituitary gland is primarily regulated by corticotropin-releasing hormone from hypothalamic neurons (Herman et al., 2016). However, pharmacological evidence indicates that the ACTH release is partially mediated by βAR stimulation in the anterior pituitary (Mezey et al., 1983). Our results suggest that endogenous β1AR stimulation is necessary for the stress-induced ACTH release. The therapeutic implications of ACTH reduction in stress-related disorders warrant further investigation. Nevertheless, our findings demonstrate that systemic β1AR blockade can alleviate the elevated ACTH levels. This observation aligns with evidence suggesting that pharmacological β1AR blockade mitigates stress-induced memory impairments and anxiety in animal models (Otis et al., 2015).

### 4.3 Fyn importance for signaling adaptations in mPFC after RS

Kinase Fyn, which is primarily associated with GluN2B phosphorylation (Goebel-Goody et al., 2012), shares structural similarities with other non-receptor tyrosine kinases. Functional analyses revealed that enzyme activation requires single phosphorylation at Y416 in the SH1 domain, whereas phosphorylation at Y527, located at the carboxyl (C-) terminus of the Fyn protein, leads to kinase downregulation (Sicheri and Kuriyan, 1997; Roskoski, 2005; Vatish et al., 2009). Notably, the entire C-terminus of Fyn is involved in the negative regulation of kinase activity (Sicheri and Kuriyan, 1997), and recent studies have identified Y530 as a critical regulatory site for the conformational changes that inactivate Fyn (Cuesta-Hernández et al., 2023). Given that phosphorylation at tyrosine 530 inactivates Fyn, our findings demonstrating that chronic RS upregulates p(Y530)Fyn levels in the mPFC suggest that chronic stress inhibits Fyn activity. Evidence from Fyn-deficient mice indicates that Fyn inactivity leads to impaired spatial learning, blunted LTP in the hippocampus (Grant et al., 1992), and heightened responsiveness to fear-inducing stimuli (Miyakawa et al., 1994). In light of these findings, our data showing impaired object recognition along with altered Fyn phosphorylation in the mPFC of rats exposed to chronic RS suggest that the downregulation of Fyn activity plays a role in the mechanisms underlying stress pathology. This interpretation is supported by pharmacological studies indicating that Fyn activation is involved in the antidepressant effects of amitriptyline (Abe et al., 2019) and losartan (Diniz et al., 2018). Furthermore, decreased Src activity, a kinase closely related to Fyn, has been observed in the hippocampus in a mouse model of postpartum depression (Zhang et al., 2016). However, there is a conflicting source of evidence regarding Fyn’s role in stress pathology. Some studies suggest a therapeutic benefit from the downregulation of Fyn activity in brain structures (Wang et al., 2022). Therefore, further research is required to elucidate the precise role of Fyn activity in the mechanisms of stress-related disorders.

### 4.4 β1AR pathway engagements in RS-evoked glutamate signal disturbances in mPFC

Our results suggest that Fyn may be directly involved in the mechanism of RS-evoked downregulation of GluN2B-containing NMDA receptors in the FC. However, the impact of stress on p(Y530)Fyn levels appears to be independent of β1AR activity, as BET treatment did not influence p(Y530)Fyn levels in the mPFC. Additionally, our study revealed that 24 h after chronic RS, the activity of the cAMP-responsive element-binding protein (CREB) remains unaltered in the mPFC of RS rats. CREB is a transcription factor that regulates numerous metabolic pathways, including β-AR signaling (e.g., Glebov-McCloud et al., 2024). This finding suggests that the β1AR–cAMP–CREB pathway is not involved in the cognitive impairments induced by exposure to homotypic stress. This result is consistent with the data from studies on stress-induced cocaine-seeking behavior (Briand and Blendy, 2013), but it contradicts with findings showing downregulation of CRTC1 (a CREB coactivator) in the mPFC of animals displaying depressive-like behavior following stress exposure (Wang et al., 2020). The discrepancy between these findings may be attributed to differences in the stress pathology models used. Based on our behavioral data, our model demonstrates spatial memory impairments, whereas CREB downregulation has been reported in mice exhibiting depressive-like behavior. Another potential explanation for the lack of detectable alterations in CREB activity in the mPFC of RS rats could be the time-limited role of CREB in stress-induced intracellular signaling. We measured p/t CREB levels 24 h after the final stress session, whereas CREB-dependent changes, such as those associated with fear learning in the hippocampus (Zelek-Molik et al., 2019) or glutamate signaling alterations in the mPFC during cocaine withdrawal (Sun et al., 2013; Sun et al., 2014a; Sun et al., 2014b), have been observed primarily within the first hours following stressor exposure. To identify potential targets within glutamate signaling for the therapeutic effects of β1AR blockade, we conducted immunohistochemical analysis in the FC region. This analysis revealed the colocalization of β1AR with AMPA-R containing GluA1, predominantly in the motor cortex (M1/M2). These findings suggest that investigating the regulatory effects of β1AR blockade on AMPA-R activity altered by stress, particularly in the M1/M2 region of the FC, would be a valuable avenue for future research.

### 4.5 Limitations

The present study has several limitations. The primary limitation is that the level of glutamate receptors was assessed in a broader area of the FC, whereas Fyn and CREB levels were measured specifically in the mPFC. The second limitation is the difference in timing, as glutamate receptors and transporters were evaluated immediately after RS, whereas Fyn and CREB levels were assessed 24 h post-RS. Last, only BET was used to evaluate the therapeutic effects of β1AR blockade on stress-induced alterations in glutamate signaling within the FC. Despite these limitations, the findings of this study provide an essential foundation for future research. Specifically, they highlight the potential involvement of the M1/M2 region in stress pathology and offer insights into the precise role of Fyn activity in the mechanisms underlying stress-induced disruptions in glutamate signaling within the FC.

### 4.6 Conclusions

**FIGURE 9:**
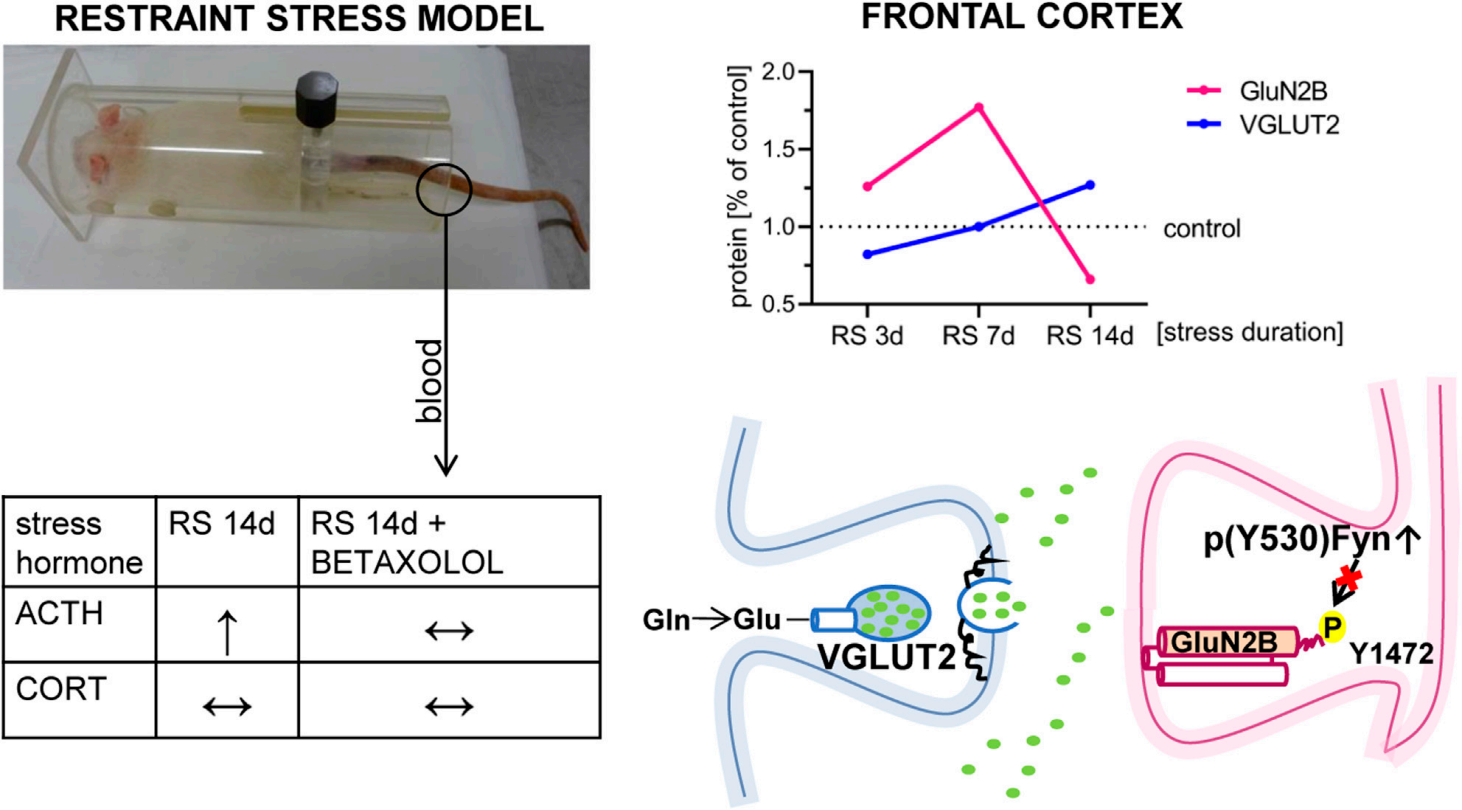
Hypothetical role of Fyn in the mechanism of GluN2B downregulation in the FC of stressed rats. Figure summarizes the results presented in the article. It depicted that RS applied for 14 days leads to a decrease in the GluN2B level in FC. GluN2B downregulation in FC, at least in the mPFC area, may be related to stress-altered Fyn activity. Although BET had no therapeutic effects on Fyn activity (see text for details), β1AR blockade normalized augmented by the stress blood level of ACTH.

Our results, summarized in Figure 9, confirm a bidirectional pattern of changes in glutamate receptor levels in the FC, which progressively develop during prolonged exposure to homotypic stress. These findings suggest that the dynamics of these changes correlate with the intensity of the applied daily stressors. The data indicate that chronic RS, accompanied by cognitive impairments, leads to a decrease in Fyn kinase activity in the mPFC, highlighting Fyn deactivation as a critical factor in the chronic stress-induced downregulation of GluN2B in the FC. Furthermore, the lack of BET impact on the assessed behavioral and biochemical parameters suggests that βAR activity is not involved in the mechanism underlying chronic RS-evoked GluN2B downregulation in the FC at least within the mPFC region.

## Data Availability

The original contributions presented in the study are included in the article/Supplementary Material; further inquiries can be directed to the corresponding author.
