# Supplementary material for: Restraint stress effects on glutamate signaling protein levels in the rats’ frontal cortex: Does β1 adrenoceptor activity matter?
Source: Front Pharmacol. 2025 Jan 6;15:1451895. doi: 10.3389/fphar.2024.1451895 (PMC11743458; doi:10.3389/fphar.2024.1451895)
Supplement: Supplementary file 1 [file Supplementaryfile1.docx]

Supplementary Material

**Restraint stress effects on glutamate signaling proteins level in the rats’ frontal cortex: Does β1 adrenoceptors activity matter?**

**Agnieszka Zelek-Molik^1^*, Anna Gądek-Michalska^2^, Michał Wilczkowski^1^, Adam Bielawski^1^, Katarzyna Maziarz^1^, Grzegorz Kreiner^1^, Irena Nalepa^1^**

^1^Department of Brain Biochemistry, ^2^Department of Physiology, Maj Institute of Pharmacology, Polish Academy of Sciences, Krakow, Poland

*** Correspondence:** Agnieszka Zelek-Molik, [zelek@if-pan.krakow.pl](mailto:zelek@if-pan.krakow.pl)

**Suplemmentary Figure 1** The lack of the influence of chronic RS and BET treatment on the rats’ behavior measured in EPM test 24 hours after last treatment. A -Time spent in open and B - closed arms of EPM apparatus, C - the frequency of entries to open and D - closed arms. White bars correspond to SHAM, black to RS pretreatment, bars with pattern correspond to BET posttreatment. BET (5 mg/kg/po/14 days) applied immediately after RS. Results are expressed as a mean ± SEM. N=7-8/group. In A: RS effect: [F(1,26)=0.16, p=0.69], BET effect [F(1.26)=0.02, p=0.89], RS×BET interaction [F(1,26)=0.11, p=0.75]; in B: RS effect [F(1,26)=0.01, p=0.94], BET effect [F(1.26)=0.46, p=0.50], RS×BET interaction [F(1,26)=0.02, p=0.87]. In C: RS effect [F(1,26)=0.39, p=0.84], BET effect [F(1.26)=0.05, p=0.82], RS×BET interaction [F(1,26)=1.32, p=0.26]; in D: RS effect [F(1,26)=0.12, p=0.73], BET effect [F(1.26)=0.15, p=0.70], RS×BET interaction [F(1,26)=0.0006, p=0.98] of EPM apparatus.
